# Migraine is not a risk factor for glaucoma: Evidence from a bidirectional Mendelian randomization study

**DOI:** 10.1097/MD.0000000000049427

**Published:** 2026-06-26

**Authors:** Zhengxiong Kou, Haiyan Zhang, Xiaofeng Hou

**Affiliations:** a Department of Neurosurgery, The People’s Hospital of Yubei District of Chongqing, Chongqing, China; b Department of Neurology, The People’s Hospital of Yubei District of Chongqing, Chongqing, China.

**Keywords:** causal relationship, glaucoma, Mendelian randomization, migraine, risk factors

## Abstract

Numerous compelling observational studies have indicated that migraine is a risk factor for the development of glaucoma. Nevertheless, some studies have observed entirely opposite results. The objective of our research is to evaluate the potential relationship between migraine and glaucoma by employing a bidirectional Mendelian Randomization (MR) approach. This method enables us to rigorously assess the causal links between these 2 conditions, thereby addressing the discrepancies in existing literature. Independent genetic variants associated with glaucoma and migraine at the genome-wide significance level were selected as instrumental variables. All summary data were obtained from the genome-wide association study database. The primary method employed in the bidirectional MR analysis was the inverse variance weighted method, while sensitivity analyses utilized the leave-one-out method, MR-Egger method, and MR-Pleiotropy RESidual Sum and Outlier‌ method. When migraine and its subtypes (specifically migraine with aura and migraine without aura) were evaluated as exposure factors, we found no evidence of a causal relationship with glaucoma and its subtypes (namely angle-closure glaucoma and open-angle glaucoma). Subsequently, in the reverse MR analysis, when glaucoma and its subtypes (angle-closure glaucoma and open-angle glaucoma) were assessed as exposure factors, there was no substantial evidence to support a causal relationship with migraine or its subtypes (migraine with aura and migraine without aura). Furthermore, sensitivity analyses also reinforced the robustness of our bidirectional MR findings. Our bidirectional MR analysis mitigates the biases associated with traditional observational studies, highlighting that there is no direct causal relationship between migraine and the risk of glaucoma.

## 1. Introduction

Migraine is primarily classified into 2 categories: migraine with aura (MA) and migraine without aura (MO).^[1]^ It is the second most common neurological disorder worldwide, affecting over 10% of the adult population and recognized as one of the leading causes of disability globally.^[2,3]^ Furthermore, migraines have significant economic and social repercussions, adversely affecting patients’ quality of life and impairing their ability to engage in work, social activities, and family life.^[4]^ The pathophysiology of migraine is a complex and multifaceted issue involving the interplay of various systems, including neurological, vascular, and inflammatory components.^[5]^ It is widely accepted that migraine attacks occur due to the activation and sensitization of the trigeminovascular system.^[6]^ This activation leads to the release of pain-producing vasoactive neuropeptides, which can result in abnormal vasoconstriction and aura symptoms when it occurs in the occipital cortex.^[7,8]^ Additionally, migraine is considered a systemic vascular disorder, associated with conditions affecting the brain, coronary arteries, retina, skin, and peripheral vascular systems.^[9]^

Glaucoma is a group of ocular diseases that affect the optic nerve, characterized by optic nerve head atrophy, cupping, and visual field defects.^[10]^ Currently, glaucoma is a leading cause of irreversible blindness worldwide, with a prevalence rate of 3.54% among adults aged 40 to 80 years. In 2020 alone, glaucoma resulted in approximately 3.6 million cases of blindness.^[11,12]^ Traditionally, elevated intraocular pressure has been identified as the most significant risk factor for glaucoma.^[13]^ However, some patients with glaucoma do not exhibit elevated eye pressure, and conversely, some individuals with elevated intraocular pressure may continue to experience disease progression even after their pressure returns to normal.^[14]^ These observations suggest that other risk factors for glaucoma exist. Recent studies have emphasized the role of vascular mechanisms in the pathophysiology of glaucoma, highlighting systemic vascular factors, such as hypertension and diabetes, as well as ocular vascular factors, including ocular blood flow and ocular perfusion pressure, which have been identified as risk factors for the disease.^[15]^

Migraine and glaucoma are common and often distressing chronic conditions with high prevalence rates in the general population. In clinical practice, it is frequently observed that these 2 diseases coexist, leading to the hypothesis that their association is not merely coincidental. Indeed, numerous compelling studies have explored the potential link between migraine and glaucoma. For instance, Lin et al found that even after adjusting for risk factors such as sex, age, monthly income, and community urbanization level, individuals with migraine had a 1.2-fold higher risk of developing glaucoma compared to those without migraine.^[16]^ Similarly, a study by Kyoung et al demonstrated an increased risk of glaucoma onset associated with migraine, particularly noting that chronic severe migraine was linked to an even higher risk.^[17]^ Supporting this, Huang et al observed a higher cumulative incidence of glaucoma in migraineurs and identified migraine as a significant risk factor for glaucoma, especially in patients under 50 years of age without comorbidities.^[18]^ Epidemiological data further substantiate this association, indicating that the prevalence of migraine among glaucoma patients (approximately 30%) is markedly higher than in non-glaucoma controls (approximately 10–15%), particularly in those with normal-tension glaucoma.^[19]^ Finally, a meta-analysis by Xu et al consolidates these observations, concluding that migraine is associated with a 24% higher risk of glaucoma compared to never having migraine.^[20]^ However, there is considerable controversy and uncertainty across different investigations. Case-control studies conducted in Chinese,^[21]^ Japanese,^[22]^ and Armenian populations,^[23]^ for instance, have reported no significant association between migraine and glaucoma. One possible reason for this is that in observational epidemiological studies, even when a strong statistical correlation is observed, it is rare to establish a definitive causal relationship.^[24]^ Previous studies investigating the association between migraine and glaucoma have predominantly been observational in nature, leading to the perception that this relationship is tenuous. The findings from observational studies may be subject to bias due to residual confounding factors and reverse causality, which can influence the results.

Mendelian randomization (MR) analysis is an emerging epidemiological method that utilizes genetic variation as instrumental variables (IVs) to infer causal relationships from observational data.^[25]^ This approach draws on the principles of random allocation used in randomized controlled trials, as the random distribution of genotypes occurs prior to fertilization and is not influenced by individual behaviors or environmental factors. Consequently, MR analysis effectively mitigates biases arising from confounding and reverse causation issues inherent in observational epidemiological studies, thereby providing more reliable evidence.^[26]^ MR analysis has been widely utilized for causal inference between various diseases, such as the potential relationship between migraine and stroke,^[27]^ as well as between glaucoma and rheumatic diseases.^[12]^ Therefore, in this study, our objective is to utilize the most recent and largest genome-wide association studies (GWAS) on migraine and glaucoma to ascertain the causal relationship between these 2 conditions, aiming to yield new insights.

## 2. Materials and method

### 2.1. Study design

**Figure 1.**
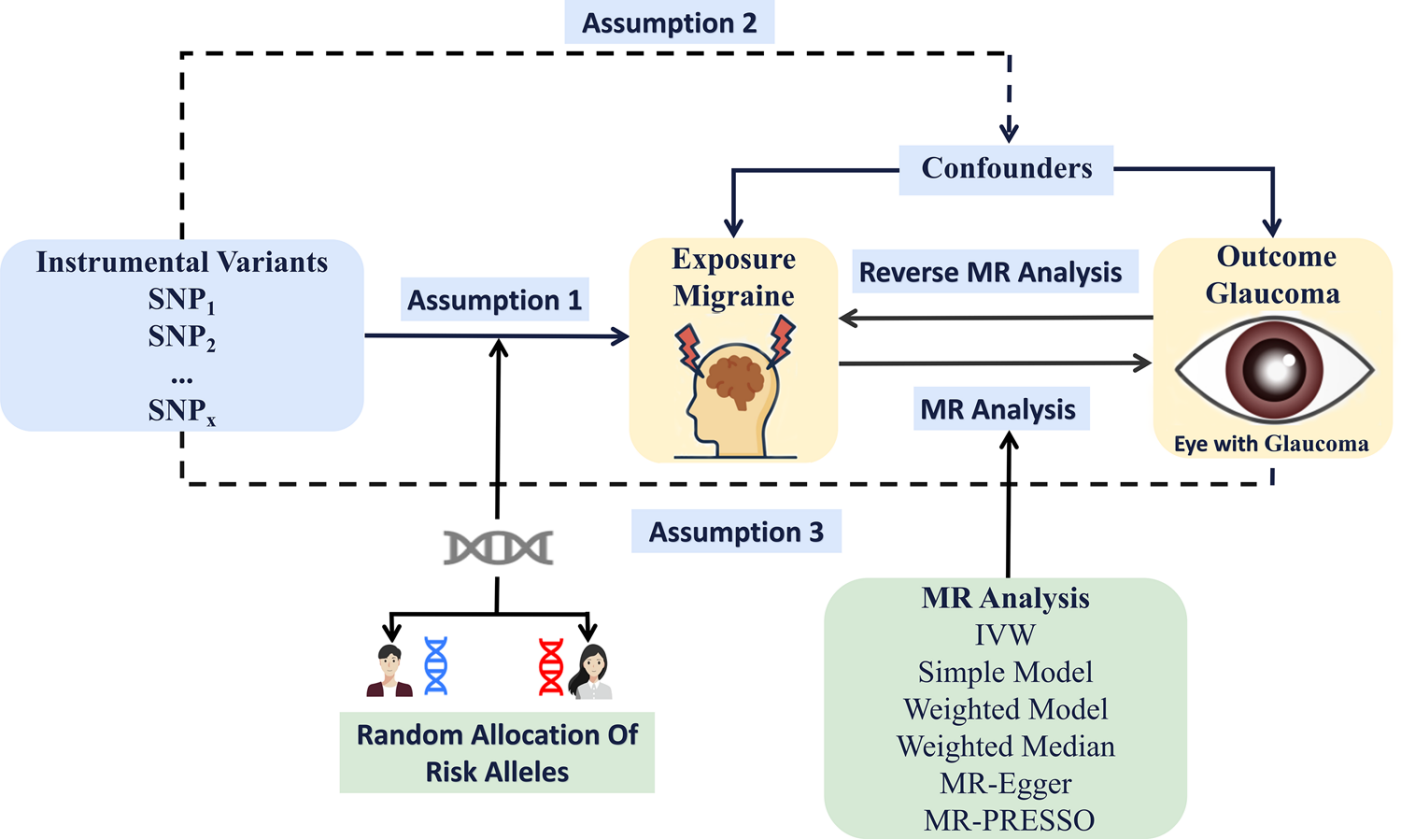
A framework design for bidirectional MR analyses. Assumption 1: Genetic variants are associated with exposure. Assumption 2: Genetic variants are not associated with any known or unknown confounders. Assumption 3: Genetic variants should only affect the risk of outcome through exposure. IVW = inverse-variance weighted, SNP = single nucleotide polymorphism.

Figure 1 illustrates the framework utilized in this study. Essentially, we employed GWAS data related to migraine (glaucoma) and its subtypes as exposure variables, while the datasets associated with glaucoma (migraine) and its subtypes served as outcome variables for a bidirectional MR analysis. Following stringent inclusion and exclusion criteria, single nucleotide polymorphisms (SNPs) associated with migraine (glaucoma) and its subtypes were selected as IVs. To minimize population stratification bias, both the exposure and outcome cohorts were restricted to individuals of European ancestry. Robust MR analysis design relies on 3 fundamental assumptions. First, IV assumption. The selected genetic variations (such as SNPs) must be significantly associated with the exposure variable. This implies that these genetic variations can influence the level of exposure in individuals. Second, no confounding. The association between the genetic variation and the outcome variable should not be affected by confounding factors. In other words, any relationship between the genetic variation and the outcome variable must operate through the exposure variable, without being influenced by other potential confounders. Third, no selection bias. The effects of genetic variation should be random across all individuals and not influenced by selection mechanisms. This ensures the representativeness of the sample, indicating that the genetic variations are unrelated to participant selection or survival status. These assumptions form the foundation for ensuring that the results of MR studies are reliable and valid. When these assumptions are met, MR can provide strong evidence for causal inference.

### 2.2. Data sources

Detailed information regarding the datasets related to all migraine and glaucoma is presented in Table 1. The genetic association data for migraine and glaucoma were obtained from the FinnGen database (http://www.finngen.fi), which includes estimated genotype data from up to 260,405 individuals of Finnish ancestry.

**Table 1:** Exposures and outcomes of the GWAS in this study.

Exposures	Dataset	Sample size (cases/ controls)	nSNPs	Population	Year
Migraine	finn-b-G6_MIGRAINE	184,654 (8547/176,107)	16,380,367	European	2021
MA	finn-b-G6_MIGRAINE_WITH_AURA	179,648 (3541/176,107)	16,380,353	European	2021
MO	finn-b-G6_MIGRAINE_NO_AURA	179,322 (3215/176,107)	16,380,336	European	2021
Glaucoma	finn-b-H7_GLAUCOMA	218,792 (8591/210,201)	16,380,466	European	2021
OAG	finn-b-H7_GLAUCPRIMOPEN	214,634(4433/210,201)	16,380,455	European	2021
ACG	finn-b-H7_GLAUCCLOSEPRIM	210,789 (588/210,201)	16,380,446	European	2021

ACG = angle-closure glaucoma, GWAS = genome-wide association study, IVW = inverse-variance weighted, MA = migraine with aura, MO = migraine without aura, MR = Mendelian randomization, nSNP = number of single nucleotide polymorphism, OAG = open-angle glaucoma.

### 2.3. IV selection

First, to satisfy the core assumption of IV analysis, our study employed a significance threshold of *P* < 5 × 10^−8^ for genome-wide exposure. However, only glaucoma (finn-b-H7_GLAUCOMA) and primary open-angle glaucoma (OAG) (finn-b-H7_GLAUCPRIMOPEN) yielded a sufficient number of SNPs for selection. Previous research has indicated that even when using a screening threshold of *P* < 5 × 10^−6^, the likelihood of weak instrument bias arising in MR analysis during linear regression of each genetic variant on the exposure variable is low.^[28]^ Secondly, we set thresholds of *R*^2^ < 0.001 and kb > 10 Mb to filter the datasets, ensuring that each exposure generated a set of independent variants. We also excluded palindromic SNPs to ensure that the alleles exerted consistent effects on both the exposure and the outcome. The *F*-statistic is used to assess the bias introduced by the IVs. It is calculated using the equation *F* = (*R*^2^ × (n−2))/ (1−*R*^2^) to evaluate the strength of the IVs and the correlation between the exposure and the outcome. A value of *F* ≥ 10 indicates a significant association. The estimated *R*^2^ for the IVs is calculated using the equation 2 EAF (1 - EAF) * *β*^2^, where effect allele frequency (EAF) represents the frequency of the effect allele and *β* denotes the estimated genetic effect on the exposure variable.^[29]^ Table S1, Supplemental Digital Content 1 provides specific SNP information along with corresponding *R*^2^ and *F*-statistics. The PhenoScanner database (https://www.repository.cam.ac.uk/items/31e4df31-982b-452e-baf8-83ce942b1c0a) was used to exclude all known phenotypes associated with any genetic instruments considered in our analysis.^[30]^

### 2.4. Bidirectional MR analysis of migraine and glaucoma

First, we examined whether migraine influences the risk of developing glaucoma, followed by a reverse MR analysis to assess whether glaucoma affects the incidence of migraine (bidirectional causal relationship). For each step of this MR study design, we employed random effects inverse variance weighted (IVW) as the primary analysis method. This approach accounts for potential heterogeneity in the causal estimates of specific variants. The results were converted to ORs expressed per genetically predicted 1-unit-higher log-odds/per unit change/per standard deviation of liability to the exposure.^[31]^ We also employed additional methods, including the weighted median method, simple mode method, weighted mode method, and MR-Egger regression, to rigorously examine the causal relationships.^[32]^ These complementary analyses were conducted to enhance the reliability and accuracy of our study findings.

### 2.5. Sensitivity analysis

First, to determine the presence of pleiotropy in the IVs and its potential impact on the results, we conducted sensitivity analyses. Cochran *Q* test was employed to assess SNP heterogeneity, with a *P* value < .05 indicating the presence of heterogeneity. Secondly, we utilized the MR-Egger intercept test to detect the possibility of horizontal pleiotropic effects, providing evidence of potential violations of the exclusion restriction (*P* < .05). MR- awas also applied to systematically evaluate the impact of pleiotropy through global testing. Then, a leave-one-out analysis was performed to assess the dependence of the MR results on each IV. Finally, we plotted funnel plots to evaluate the symmetry of the SNPs and assess the reliability of the results.

### 2.6. Statistical analysis

All statistical analyses were conducted using the R package“TwoSampleMR” (version 0.5.6) in R (version 4.2.3; MRC Integrated Epidemiology Unit [MRC IEU], University of Bristol). For more detailed instructions, please refer to the following link: https://mrcieu.github.io/TwoSampleMR/articles/gwas2020.html. The results are presented as odds ratios (OR) with 95% confidence intervals (95% CI) to quantify the magnitude of the causal relationship between migraine and glaucoma. Statistical significance for determining the causal relationship between migraine and glaucoma was set at a *P* value of < .05.

### 2.7. Ethical review

MR studies utilize existing genetic data from GWAS for analysis, thereby eliminating the need for additional ethical approvals.

## 3. Results

### 3.1. Bidirectional MR Analysis Results

**Figure 2.**
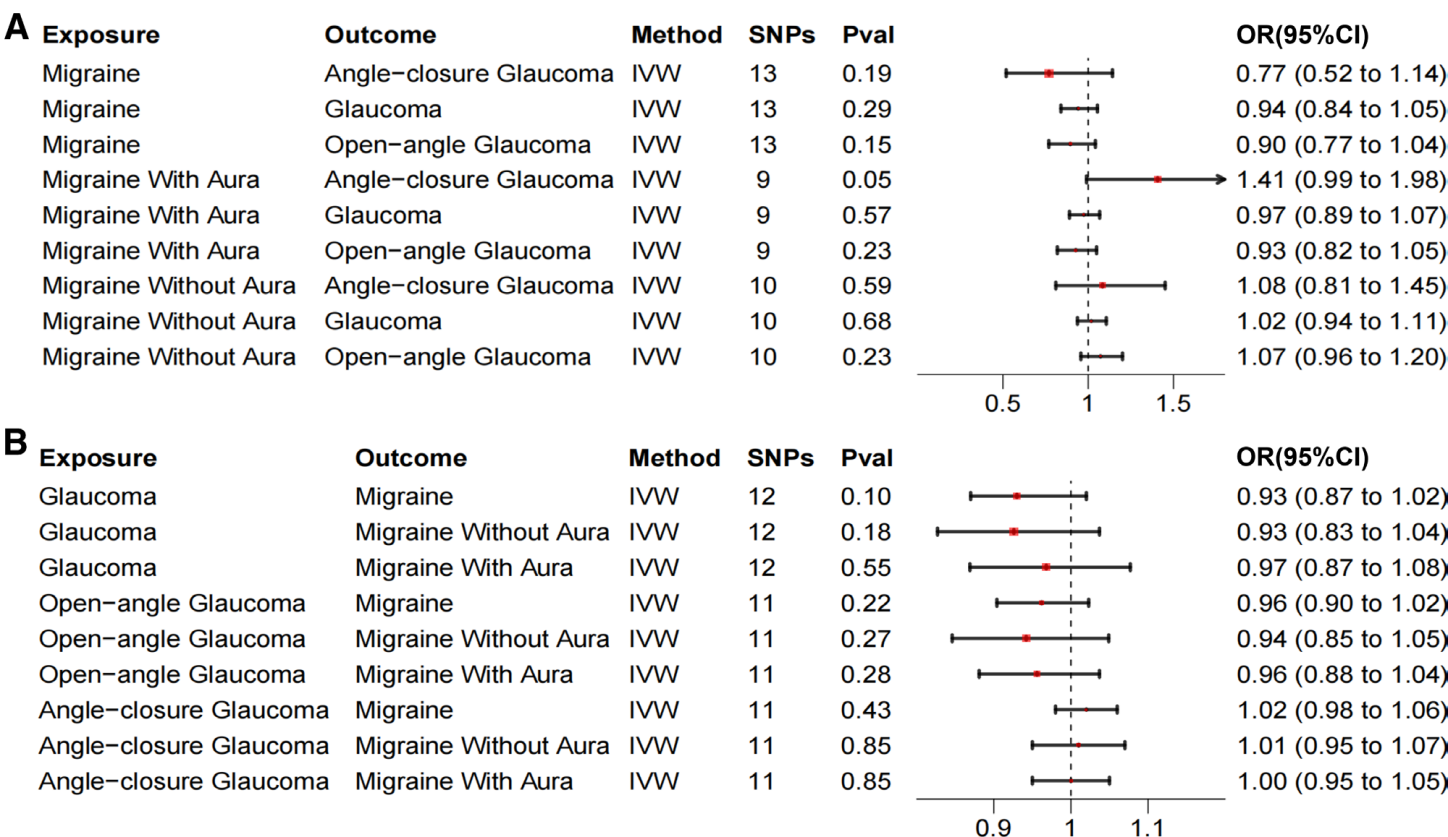
Bidirectional MR analysis of the causal relationship between migraine and glaucoma. CI = confidenc interval, IVW = inverse-variance weighted, OR = odds ratio, SNP = single nucleotide polymorphism.

**Figure 3.**
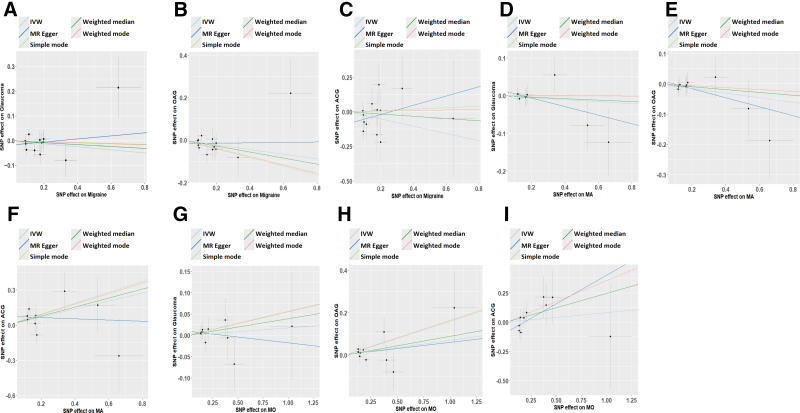
Scatter plots of SNPs associated with migraine and its subtypes and glaucoma and its subtypes. Each black point representing each SNP on the exposure (horizontal-axis) and on the outcome (vertical-axis) is plotted with error bars corresponding to each SE. The MR regression slopes of the lines represent the causal estimates using 5 approaches. (A) Migraine and glaucoma; (B) Migraine and OAG; (C) Migraine and ACG; (D) MA and glaucoma; (E) MA and OAG; (F) MA and ACG; (G) MO and glaucom; (H) MO and OAG; (I) MO and ACG. ACG = angle-closure glaucoma, IVW = inverse-variance weighted, MA = migraine with aura, MO = migraine without aura, MR = Mendelian randomization, OAG = open-angle glaucoma, SE = standard error, SNP = single nucleotide polymorphism.

**Figure 4.**
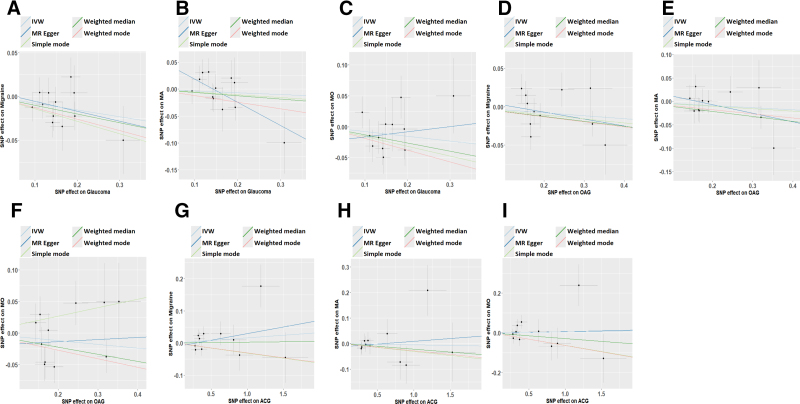
Scatter plots of SNPs associated with glaucoma and its subtypes and migraine and its subtypes. Each black point representing each SNP on the exposure (horizontal-axis) and on the outcome (vertical-axis) is plotted with error bars corresponding to each SE. The MR regression slopes of the lines represent the causal estimates using 5 approaches. (A) Glaucoma and migraine; (B) Glaucoma and MA; (C) Migraine and MO; (D) OAG and glaucoma; (E) OAG and MA; (F) OAG and MO; (G) ACG and glaucoma; (H) ACG and MA; (I) ACG and MA. ACG = angle-closure glaucoma, IVW = inverse-variance weighted, MA = migraine with aura, MO = migraine without aura, MR = Mendelian randomization, OAG = open-angle glaucoma, SE = standard error, SNP = single nucleotide polymorphism.

MR analysis employs genetic variations to determine whether the observed associations between risk factors and outcomes are consistent with causal relationships. The *F*-statistics for all SNPs included in the analysis were > 10 (Table S1, Supplemental Digital Content 1). After assessing and removing SNPs associated with confounding, the results of the bidirectional 2-sample MR analysis examining the relationship between migraine and its subtypes with glaucoma and its subtypes were evaluated using the IVW method, as presented in Figure 2. To provide a more visual representation of the MR analysis results, we also plotted scatter plots (Figs. 3 and 4). As shown in Figure 2, our forward MR analysis (overall migraine as exposure) using the IVW method observed no causal effect of overall migraine on overall glaucoma (OR = 0.94, 95% CI = 0.84–1.05, *P* = .29), OAG (OR = 0.90, 95% CI = 0.77–1.04, *P* = .15), or angle-closure glaucoma (ACG) (OR = 0.77, 95% CI = 0.52–1.14, *P* = .19). Additionally, when migraine subtypes were examined as independent exposures, neither MO nor MA showed a significant causal effect on the risk of overall glaucoma, OAG, or ACG (all *P* > .05). The full set of ORs and CIs for these subtype-specific analyses is presented in Figure 2. Similarly, in reverse MR analyses, neither overall glaucoma nor its subtypes (OAG, ACG) demonstrated a causal effect on overall migraine or its subtypes (Fig. 2).

### 3.2. Sensitivity analysis

**Figure 5.**
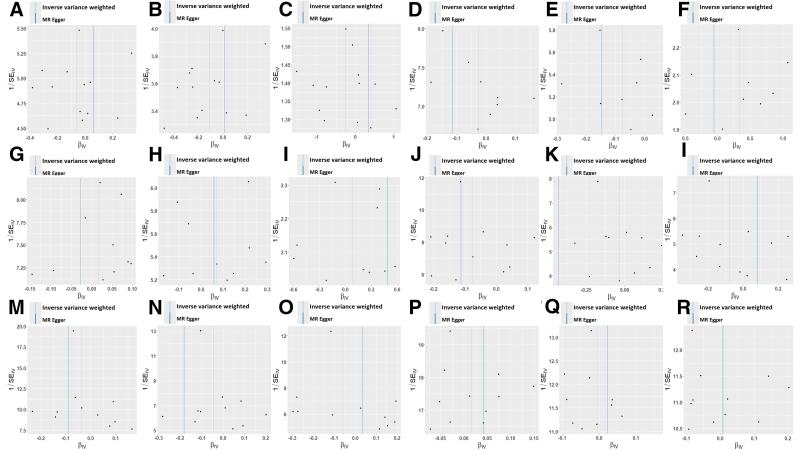
Funnel plot showing the relationship between the cause-effect of glaucoma and its subtypes and migraine and its subtypes. (A) Migraine and glaucoma; (B) Migraine and OAG; (C) Migraine and ACG; (D) MA and glaucoma; (E) MA and OAG; (F) MA and ACG; (G) MO and glaucoma; (H) MO and OAG; (I) MO and ACG; (J) Glaucoma and migraine; (K) Glaucoma and MA; (L) Migraine and MO; (M) OAG and glaucoma; (N) OAG and MA; (O) OAG and MO; (P) ACG and glaucoma; (Q) ACG and MA; (R) ACG and MA. ACG = angle-closure glaucoma, IVW = inverse-variance weighted, MA = migraine with aura, MO = migraine without aura, MR = Mendelian randomization, OAG = open-angle glaucoma, SE = standard error, SNP = single nucleotide polymorphism.

**Figure 6.**
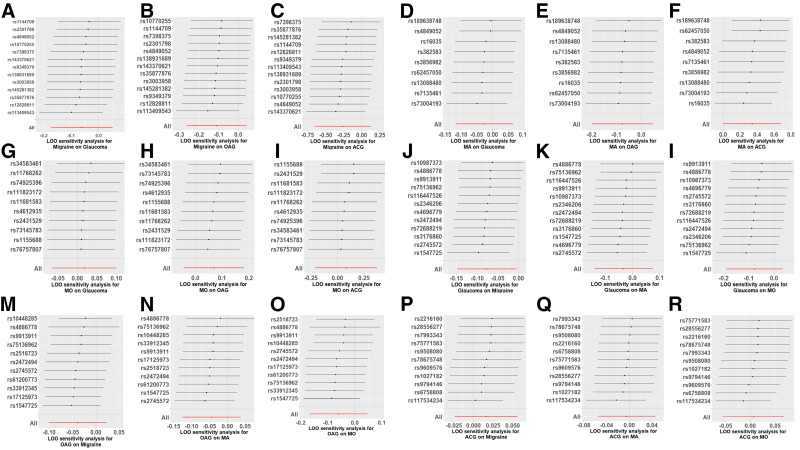
Forest plots of LOO analyses for causal SNP effects of glaucoma and its subtypes and migraine and its subtypes. (A) Migraine and glaucoma; (B) Migraine and OAG; (C) Migraine and ACG; (D) MA and glaucoma; (E) MA and OAG; (F) MA and ACG: (G) MO and glaucoma; (H) MO and OAG; (I) MO and ACG;. (J) Glaucoma and migraine;. (K) Glaucoma and MA; (L) Migraine and MO; (M) OAG and glaucoma; (N) OAG and MA; (O) OAG and MO; (P) ACG and glaucoma; (Q) ACG and MA; (R) ACG and MA. ACG = angle-closure glaucoma, IVW = inverse-variance weighted, LOO = leave-one-out, MA = migraine with aura, MO = migraine without aura, MR = Mendelian randomization, OAG = open-angle glaucoma, SE = standard error, SNP = single nucleotide polymorphism.

We conducted sensitivity analyses separately for both MR analysis and reverse MR analysis. The results of the Cochran *Q* statistic, MR-Egger intercept test, and MR-PRESSO test are presented in Table 2. Both MR-Egger and IVW methods did not show significant heterogeneity in Cochran *Q* test (*P* > .05). Similarly, all *P* values from the MR-Egger intercept test and MR-PRESSO test were > .05, indicating the absence of horizontal pleiotropy. The funnel plot demonstrated a symmetrical distribution of SNPs, underscoring the relative stability of the results (Fig. 5). Additionally, leave-one-out analysis revealed that the associations between migraine and its subtypes and glaucoma and its subtypes were not driven by any single SNP; this was also observed in the reverse MR analysis (Fig. 6). These findings further corroborate the robustness of our MR analysis results.

**Table 2:** Pleiotropy and heterogeneity test.

Exposure	Outcome	MR-Egger intercept test		Cochran *Q* test				MR-PRESSO	
		Egger_intercept	*P* val	*Q* (IVW)	*Q*_*P* val	Q (MR-Egger)	*Q*_*P*val	Outlier	*P* val
Migraine	Glaucoma	−0.0175	.35	12.59	.39	11.61	.39	14.86	.38
Migraine	OAG	−0.0172	.49	0.78	7.97	7.46	.76	9.41	.78
Migraine	ACG	−0.0846	.20	12.13	.43	10.29	.50	14.20	.47
MA	Glaucoma	0.0166	.37	4.69	.78	3.81	.80	5.99	.81
MA	OAG	0.0135	.59	2.58	.95	2.27	.94	3.28	.96
MA	ACG	0.0745	.29	9.29	.31	7.85	.34	11.76	.35
MO	Glaucoma	0.0095	.59	3.03	.96	2.73	.95	3.70	.97
MO	OAG	0.0024	.91	6.56	.68	6.55	.58	8.14	.70
MO	ACG	−0.0871	.19	7.18	.61	5.16	.73	8.84	.62
Glaucoma	Migraine	0.0056	.79	7.47	.75	7.40	.68	8.93	.79
Glaucoma	MA	0.0616	.07	10.29	.50	6.50	.77	12.51	.46
Glaucoma	MO	−0.0248	.48	10.95	.44	10.40	.40	13.27	.44
OAG	Migraine	0.0112	.63	12.83	.23	12.50	.18	15.07	.28
OAG	MA	0.0298	.33	7.66	.66	6.60	.67	9.38	.68
OAG	MO	−0.0202	.62	15.65	.11	15.21	.08	18.79	.11
ACG	Migraine	−0.0119	.56	14.15	.16	13.61	.13	17.15	.17
ACG	MA	−0.0127	.61	8.61	.56	8.33	.50	10.4	.56
ACG	MO	−0.0009	.97	13.56	.19	13.56	.13	16.50	.19

ACG = angle-closure glaucoma, IVW = inverse-variance weighted, MA = migraine with aura, MO = migraine without aura, MR = Mendelian randomization, OAG = open-angle glaucoma.

## 4. Discussion

Migraine and glaucoma are both highly prevalent conditions, and a growing number of observational studies have suggested a possible association between them.^[33]^ However, the nature of this relationship (whether causal or coincidental) remains controversial due to inconsistent findings across studies.^[20]^ In this study, we employed a bidirectional MR approach using large-scale GWAS data to investigate the potential causal link between migraine and glaucoma. Our primary finding is that there is no evidence of a bidirectional causal relationship between migraine (including its subtypes MA and MO) and glaucoma (including OAG and ACG). This suggests that the previously reported epidemiological associations may be attributable to confounding factors or shared underlying mechanisms rather than a direct causal pathway. Notably, the MR design used here offers several methodological strengths that enhance the robustness of our conclusions. First, by using genetic variants as IVs, MR minimizes the influence of unmeasured confounding and environmental factors that often plague observational studies. Second, the bidirectional approach allows us to examine causality in both directions, effectively ruling out reverse causation as an explanation for the observed associations. Third, the consistency of results across multiple sensitivity analyses (including MR-Egger, MR-PRESSO, and leave-one-out tests) supports the reliability of our null findings and suggests the absence of significant horizontal pleiotropy.^[34,35]^

Some epidemiological studies have reported that individuals with migraine exhibit a higher prevalence of glaucoma, particularly OAG.^[17]^ For instance, studies based on European populations have suggested that migraine is associated with a 20 to 30% increased risk of glaucoma, with a reported prevalence of migraine among glaucoma patients ranging from approximately 30%, compared to 10 to 15% in non-glaucoma controls.^[19]^ However, these estimates vary considerably across different populations and study designs. For example, studies in Asian populations have often failed to replicate these associations, highlighting potential geographical and ethnic heterogeneity.^[21,22]^ Such variability underscores the challenges of drawing causal inferences from observational data alone and may explain why some studies report positive associations while others do not.

Indeed, despite the epidemiological overlap, our MR analysis did not support migraine as a causal risk factor for glaucoma, nor did it indicate that glaucoma predisposes individuals to migraine. This aligns with several prior studies that have questioned the strength and consistency of the migraine-glaucoma link.^[21–23]^ It is worth noting that migraine and glaucoma may share certain biological mechanisms, as both conditions are associated with neurovascular regulation and involve vascular changes in the brain and eyes. Retinal vascular dysregulation and impaired blood flow to the optic nerve head are considered risk factors for the development of glaucoma.^[36]^ Similarly, migraine is defined as a pathophysiological condition that simultaneously involves vascular and neurological components.^[37]^ The pain of migraine is triggered by the activation of the trigeminovascular system.^[38]^ Additionally, certain neurotransmitters and inflammatory mediators, such as calcitonin gene-related peptide, may play a role in the pathological processes of both migraine and glaucoma, with abnormal fluctuations in these mediators leading to vascular dysfunction.^[39,40]^ Some studies even suggest that the potential link between migraine and glaucoma may be attributed to a shared mechanism of vascular spasm.^[41]^ Genetic research has also revealed multiple gene variants associated with both migraine and glaucoma, indicating that certain genetic factors may establish a connection between the 2 diseases. For example, several genes related to vascular regulation and neural function, such as matrix metalloproteinases, have been shown to be expressed in both conditions.^[42,43]^ Unfortunately, these studies have attempted to establish a potential link between migraine and glaucoma through numerous hypotheses and theories; however, there is currently no substantial evidence to indicate that these commonalities necessarily translate into a direct causal relationship.

The connection between migraine and glaucoma remains an active area of research. Our study emphasizes the absence of a causal relationship between migraine and glaucoma by utilizing MR analysis, an emerging epidemiological tool. This study has several noteworthy advantages. Firstly, it is the first investigation of the causal relationship between migraine and glaucoma. Traditional observational studies are often affected by reverse causation and confounding factors, which diminishes their reliability when making causal inferences. The MR analysis design employed in this study effectively mitigates the risks of reverse causation and most confounding factors. Furthermore, compared to conventional studies, MR research is more convenient, cost-effective, and less labor-intensive. It is important to note, however, that we identified several limitations in this study. Firstly, our research primarily focused on individuals of European ancestry, which restricts the generalizability of our findings. Future studies should include more diverse populations to validate these findings across different ethnic groups. Secondly, while this study provides sufficient evidence to draw compelling conclusions, further research will help deepen our understanding of this relationship.

In summary, our bidirectional MR analysis found no evidence of a causal relationship between migraine (including its subtypes MA and MO) and glaucoma (including OAG and ACG). This suggests that the association observed in prior epidemiological studies is unlikely to be causal. The study highlights the value of genetic approaches in clarifying such disputed links, indicating that migraine and glaucoma likely develop via independent pathways.

## Acknowledgments

The authors thank the FinnGen study in our analysis for providing a publicly available GWAS dataset.

## Author contributions

**Conceptualization:** Zhengxiong Kou, Haiyan Zhang, Xiaofeng Hou.

**Data curation:** Zhengxiong Kou, Haiyan Zhang, Xiaofeng Hou.

**Investigation:** Zhengxiong Kou, Haiyan Zhang, Xiaofeng Hou.

**Project administration:** Xiaofeng Hou.

**Writing – original draft:** Zhengxiong Kou, Haiyan Zhang, Xiaofeng Hou.

**Writing – review & editing:** Zhengxiong Kou, Haiyan Zhang, Xiaofeng Hou.



## References

[1] Arnold MJC. Headache Classification Committee of the International Headache Society (IHS) the international classification of headache disorders, 3rd edition. Cephalalgia. 2018;38:1–211. 10.1177/0333102417738202 29368949

[2] GBD 2016 Neurology Collaborators. Global, regional, and national burden of neurological disorders, 1990-2016: a systematic analysis for the global burden of disease study 2016. Lancet Neurol. 2019;18:459–80. 30879893 10.1016/S1474-4422(18)30499-X PMC6459001

[3] Stovner L Hagen K Jensen R. The global burden of headache: a documentation of headache prevalence and disability worldwide. Cephalalgia. 2007;27:193–210. 17381554 10.1111/j.1468-2982.2007.01288.x

[4] Lipton RB Hamelsky SW Kolodner KB Steiner TJ Stewart WF. Migraine, quality of life, and depression: a population-based case-control study. Neurology. 2000;55:629–35. 10980724 10.1212/wnl.55.5.629

[5] Zagami AS. Pathophysiology of migraine and tension-type headache. Curr Opin Neurol. 1994;7:272–7. 8081522 10.1097/00019052-199406000-00016

[6] Cui Y Kataoka Y Watanabe Y. Role of cortical spreading depression in the pathophysiology of migraine. Neurosci Bull. 2014;30:812–22. 25260797 10.1007/s12264-014-1471-y PMC5562594

[7] Russo A Tessitore A Tedeschi G. Migraine and trigeminal system-I can feel it coming…. Curr Pain Headache Rep. 2013;17:367. 23996723 10.1007/s11916-013-0367-2

[8] Sarrouilhe D Dejean C Mesnil M. Involvement of gap junction channels in the pathophysiology of migraine with aura. Front Physiol. 2014;5:78. 24611055 10.3389/fphys.2014.00078 PMC3933780

[9] Charles A. The pathophysiology of migraine: implications for clinical management. Lancet Neurol. 2018;17:174–82. 29229375 10.1016/S1474-4422(17)30435-0

[10] Cohen LP Pasquale LR. Clinical characteristics and current treatment of glaucoma. Cold Spring Harb Perspect Med. 2014;4:a017236. 24890835 10.1101/cshperspect.a017236 PMC4031956

[11] Tham YC Li X Wong TY Quigley HA Aung T Cheng C-Y. Global prevalence of glaucoma and projections of glaucoma burden through 2040: a systematic review and meta-analysis. Ophthalmology. 2014;121:2081–90. 24974815 10.1016/j.ophtha.2014.05.013

[12] Meng Y Tan Z Su Y Li L Chen C. Causal association between common rheumatic diseases and glaucoma: a Mendelian randomization study. Front Immunol. 2023;14:1227138. 37799717 10.3389/fimmu.2023.1227138 PMC10550209

[13] Stein JD Khawaja AP Weizer JS. Glaucoma in adults-screening, diagnosis, and management: a review. JAMA. 2021;325:164–74. 33433580 10.1001/jama.2020.21899

[14] Funk RO Hodge DO Kohli D Roddy GW. Multiple systemic vascular risk factors are associated with low-tension glaucoma. J Glaucoma. 2022;31:15–22. 34731871 10.1097/IJG.0000000000001964 PMC9337264

[15] Van Eijgen J Melgarejo JD Van Laeken J. The relevance of arterial blood pressure in the management of glaucoma progression: a systematic review. Am J Hypertens. 2024;37:179–98. 37995334 10.1093/ajh/hpad111 PMC10906067

[16] Lin HC Chien C-W Hu C-C Ho J-D. Comparison of comorbid conditions between open-angle glaucoma patients and a control cohort: a case-control study. Ophthalmology. 2010;117:2088–95. 20570357 10.1016/j.ophtha.2010.03.003

[17] Ohn K Han K Moon JI Jung Y. Presence and severity of migraine is associated with development of primary open angle glaucoma: a population-based longitudinal cohort study. PLoS One. 2023;18:e0283495. 36961849 10.1371/journal.pone.0283495 PMC10038297

[18] Huang JY Su C-C Wang T-H Tsai I-J. Migraine and increased risk of developing open angle glaucoma: a population-based cohort study. BMC Ophthalmol. 2019;19:50. 30760249 10.1186/s12886-019-1062-9 PMC6375150

[19] Nguyen BN Lek JJ Vingrys AJ McKendrick AM. Clinical impact of migraine for the management of glaucoma patients. Prog Retin Eye Res. 2016;51:107–24. 26232725 10.1016/j.preteyeres.2015.07.006

[20] Xu C Li J Li Z Mao X. Migraine as a risk factor for primary open angle glaucoma: A systematic review and meta-analysis. Medicine (Baltimore). 2018;97:e11377. 29995778 10.1097/MD.0000000000011377 PMC6076184

[21] Lin HC Kang J-H Jiang Y-D Ho J-D. Hypothyroidism and the risk of developing open-angle glaucoma: a five-year population-based follow-up study. Ophthalmology. 2010;117:1960–6. 20557938 10.1016/j.ophtha.2010.02.005

[22] Usui T Iwata K Shirakashi M Abe H. Prevalence of migraine in low-tension glaucoma and primary open-angle glaucoma in Japanese. Br J Ophthalmol. 1991;75:224–6. 2021590 10.1136/bjo.75.4.224 PMC1042327

[23] Giloyan A Khachadourian V Hakobyan V Kirakosyan L Petrosyan V Harutyunyan T. Migraine headache and other risk factors associated with glaucoma among the adult population living in Armenia: a case-control study. Int Ophthalmol. 2024;44:188. 38647698 10.1007/s10792-024-03145-2

[24] Bochud M. On the use of Mendelian randomization to infer causality in observational epidemiology. Eur Heart J. 2008;29:2456–7. 18812324 10.1093/eurheartj/ehn428

[25] Ziegler A Pahlke F König IR. Comments on “Mendelian randomization: using genes as instruments for making causal inferences in epidemiology” by Debbie A. Lawlor, R. M. Harbord, J. A. Sterne, N. Timpson and G. Davey Smith, Statistics in Medicine, DOI: 10.1002/sim.3034. Stat Med. 2008;27:2974–6; author reply 2976. 18203119 10.1002/sim.3213

[26] Lawlor DA Harbord RM Sterne JAC Timpson N Davey Smith G. Mendelian randomization: using genes as instruments for making causal inferences in epidemiology. Stat Med. 2008;27:1133–63. 17886233 10.1002/sim.3034

[27] Shu MJ Li J-R Zhu Y-C Shen H. Migraine and ischemic stroke: a mendelian randomization study. Neurol Ther. 2022;11:237–46. 34904213 10.1007/s40120-021-00310-y PMC8857343

[28] Burgess S Butterworth A Thompson SG. Mendelian randomization analysis with multiple genetic variants using summarized data. Genet Epidemiol. 2013;37:658–65. 24114802 10.1002/gepi.21758 PMC4377079

[29] Chen J Wu C He J. Causal associations of thyroid function and sudden sensorineural hearing loss: a bidirectional and multivariable Mendelian randomization study. Front Neurol. 2023;14:1269545. 38090267 10.3389/fneur.2023.1269545 PMC10715417

[30] Kamat MA Blackshaw JA Young R. PhenoScanner V2: an expanded tool for searching human genotype-phenotype associations. Bioinformatics. 2019;35:4851–3. 31233103 10.1093/bioinformatics/btz469 PMC6853652

[31] Pierce BL Burgess S. Efficient design for Mendelian randomization studies: subsample and 2-sample instrumental variable estimators. Am J Epidemiol. 2013;178:1177–84. 23863760 10.1093/aje/kwt084 PMC3783091

[32] Hemani G. The MR-Base platform supports systematic causal inference across the human phenome. Elife. 2018;7:e34408. 29846171 10.7554/eLife.34408 PMC5976434

[33] Wang JJ Mitchell P Smith W. Is there an association between migraine headache and open-angle glaucoma? Findings from the Blue Mountains Eye Study. Ophthalmology. 1997;104:1714–9. 9331214 10.1016/s0161-6420(97)30075-x

[34] Emdin CA Khera AV Kathiresan S. Mendelian randomization. JAMA. 2017;318:1925–6. 29164242 10.1001/jama.2017.17219

[35] Sekula P Del Greco M F Pattaro C Köttgen A. Mendelian randomization as an approach to assess causality using observational data. J Am Soc Nephrol. 2016;27:3253–65. 27486138 10.1681/ASN.2016010098 PMC5084898

[36] Gramer G Weber BH Gramer E. Migraine and vasospasm in glaucoma: age-related evaluation of 2027 patients with glaucoma or ocular hypertension. Invest Ophthalmol Vis Sci. 2015;56:7999–8007. 26720447 10.1167/iovs.15-17274

[37] Charles A. The evolution of a migraine attack - a review of recent evidence. Headache. 2013;53:413–9. 23278169 10.1111/head.12026

[38] NosedaRBursteinR. Migraine pathophysiology: anatomy of the trigeminovascular pathway and associated neurological symptoms, cortical spreading depression, sensitization, and modulation of pain. Pain. 2013;154:S44–53.23891892 10.1016/j.pain.2013.07.021

[39] Vesaluoma M Mertaniemi P Mannonen S. Cellular and plasma fibronectin in the aqueous humour of primary open-angle glaucoma, exfoliative glaucoma and cataract patients. Eye (Lond). 1998;12 (Pt 5):886–90. 10070530 10.1038/eye.1998.224

[40] Russo AF Hay DL. CGRP physiology, pharmacology, and therapeutic targets: migraine and beyond. Physiol Rev. 2023;103:1565–644. 36454715 10.1152/physrev.00059.2021 PMC9988538

[41] Flammer J Konieczka K Flammer AJ. The primary vascular dysregulation syndrome: implications for eye diseases. EPMA J. 2013;4:14. 23742177 10.1186/1878-5085-4-14 PMC3693953

[42] Wu MY Wu Y Zhang Y. Associations between matrix metalloproteinase gene polymorphisms and glaucoma susceptibility: a meta-analysis. BMC Ophthalmol. 2017;17:48. 28431514 10.1186/s12886-017-0442-2 PMC5401566

[43] Kara I Ozkok E Aydin M. Combined effects of ACE and MMP-3 polymorphisms on migraine development. Cephalalgia. 2007;27:235–43. 17381556 10.1111/j.1468-2982.2006.01269.x

